# 231. Lacticaseibacillus rhamnosus CRL 2244 extracts as an antimicrobial strategy against Community- and Hospital-Associated Staphylococcus aureus

**DOI:** 10.1093/ofid/ofaf695.083

**Published:** 2026-01-11

**Authors:** Cecilia Rodriguez, Briea Gasca, Camila Leal, Nicholas Salzameda, Robert A Bonomo, Maria Soledad Ramirez

**Affiliations:** CERELA, Tucuman, Tucuman, Argentina; CSUF, Fullerton, California; CERELA, Tucuman, Tucuman, Argentina; CSUF, Fullerton, California; Case Western Reserve University/ Louis Stokes Cleveland VA Medical Center, Cleveland, OH; California State University Fullerton, Corona, CA

## Abstract

**Background:**

Methicillin resistant *Staphylococcus aureus* (MRSA) causes a wide range of acute and chronic infections in both hospital (HA-MRSA) and community (CA-MRSA) settings. The global rise and widespread prevalence of MRSA strains resistant to multiple antibiotic classes underscore the urgent need for novel antimicrobial therapies.

*Lacticaseibacillus rhamnosus* CRL 2244 produces bioactive compounds with potent antimicrobial activity against bacterial pathogens.

Our study investigates the efficacy of the secreted metabolites from CRL 2244 (extract) in inhibiting *S. aureus* growth, adhesion, biofilms, and enhancing antibiotic activity.

**Figure 1.**
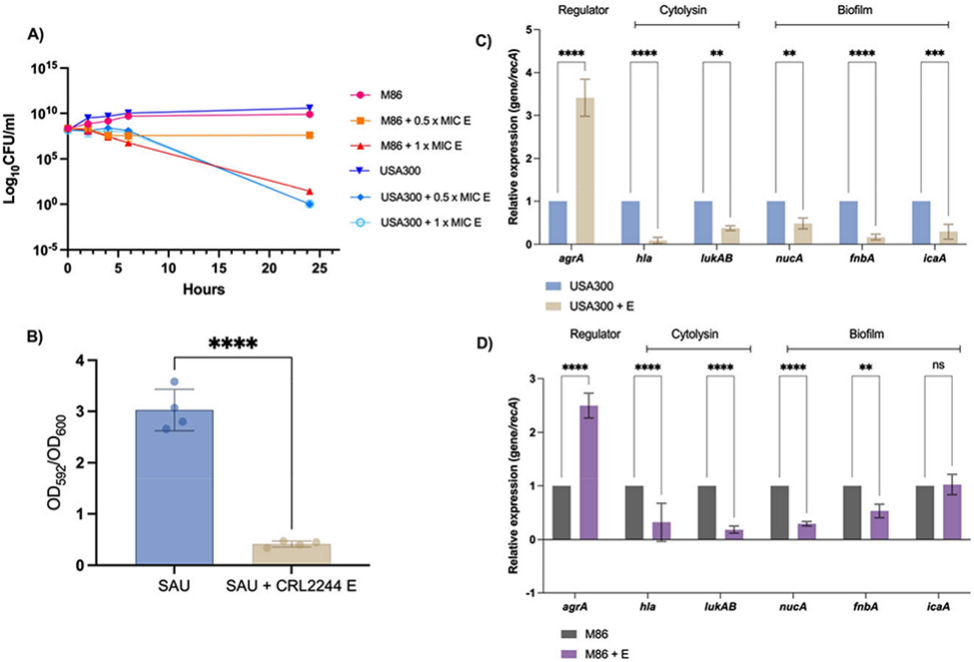
Effect of CRL2244 extract in A) killing activity against MRSA strains, B) Biofilm-forming by MRSA USA300, C) and D) Transcriptional changes of regulatory genes agrA and lukAB, hemolysin hla and biofilm-forming nucA, fnbA and icaA in USA300 and M86 strains, respectively.

**Methods:**

USA300 (CA-MRSA) and M86 (HA-MRSA) strains were used. Killing assays were used to evaluate the extract inhibitory effect. Biofilm formation was assessed using crystal violet staining assays and bacterial adhesion using coated fibronectin 96-well microplates. Synergistic effects of the extract with β-lactam antibiotics were determined by antimicrobial susceptibility assays. In addition, RNA extractions following by RT-qPCR were performed to analyze transcriptomic changes in the presence of extract.

**Results:**

The extract exhibited strong inhibitory effects against both strains at 0.5× and 1× MIC significantly reducing the bacterial survival compared to untreated controls. The effect was more pronounced at 1× MIC, particularly in the M86 strain (Fig. 1a). Biofilm assays showed that the extract significantly reduced biofilm formation, indicating its potential to weaken biofilm-associated infections (Fig.1b). The adhesion assay results indicate that adding 1x MIC the extract significantly reduced adhesion (29.40 % and 34.64 % for USA300 and M86, respectively). Synergistic assays with ampicillin and cefoxitin were seen in the presence of the extract, enhancing the efficacy of β-lactam antibiotics.

RT-qPCR analysis revealed downregulation of biofilm- and virulence-associated genes in both strains treated with the extract (Fig. 1c-d).

**Conclusion:**

Our findings demonstrate that CRL 2244 extract has the potential to be developed as an antimicrobial agent against MRSA. Its ability to inhibit bacterial growth, adhesion, disrupt biofilms, and potentiate antibiotic activity highlights its therapeutic potential.

**Disclosures:**

All Authors: No reported disclosures

